# Implementation of a CMOS/MEMS Accelerometer with ASIC Processes

**DOI:** 10.3390/mi10010050

**Published:** 2019-01-12

**Authors:** Yu-Sian Liu, Kuei-Ann Wen

**Affiliations:** Institute of Electronic Engineering, National Chiao Tung University, Hsinchu 300, Taiwan; stellawen@mail.nctu.edu.tw

**Keywords:** accelerometer design, spring design, analytical model

## Abstract

This paper presents the design, simulation and mechanical characterization of a newly proposed complementary metal-oxide semiconductor (CMOS)/micro-electromechanical system (MEMS) accelerometer. The monolithic CMOS/MEMS accelerometer was fabricated using the 0.18 μm application-specific integrated circuit (ASIC)-compatible CMOS/MEMS process. An approximate analytical model for the spring design is presented. The experiments showed that the resonant frequency of the proposed tri-axis accelerometer was around 5.35 kHz for out-plane vibration. The tri-axis accelerometer had an area of 1096 μm × 1256 μm.

## 1. Introduction

Micro-electromechanical system (MEMS) technology has enabled the substantial expansion of the inertial sensor market by decreasing power consumption, cost, and size. The complementary metal-oxide semiconductor (CMOS)/MEMS technology enables the integration of CMOS circuits with MEMS structures in a single chip [1]. CMOS/MEMS processes have the advantages of a mature foundry service for mass production, monolithic integration with CMOS circuitry to reduce the parasitic capacitance, and size reduction to decrease chip cost [2,3,4,5,6,7]. However, the composite thin-film structure of CMOS/MEMS technology suffers from residual stresses and limits the device’s performance. The CMOS/MEMS structure consisted of multiple metal and dielectric stacking layers. After release from the substrate, the structure is deformed by the thin film residual stresses, which can significantly affect the device’s performance [4,8]. The deformation of composite structures was predicted based on analytical models in [9].

A capacitive accelerometer can be implemented in the CMOS/MEMS process [10]. A capacitive CMOS/MEMS accelerometer typically consists of the proof-mass, springs, and sensing electrodes. The sensing electrodes are placed around the proof mass. The sensing technique involves using the gap-closing method [11,12]. For 3-axis integrated accelerometer design, the *z*-axis sensing element consists of an imbalanced proof mass, a torsional spring beam and comb fingers on both ends of the proof mass [13]. Using three individual sensing units to detect the tri-axis acceleration can reduce structural curling [14]. A single proof-mass tri-axis accelerometer can significantly reduce the chip size and improve the accelerometer sensitivity [15,16].

The sensitivity of accelerometers strongly depends on the spring constants of the suspension system [17]. Many types of springs can be utilized in the accelerometer design. Four commonly used flexures are: clamped-clamped flexure, crab-leg flexure, folded flexure and serpentine flexure. Among these four types, the serpentine flexure has the lowest stiffness and has reduced axial stress [18]. A serpentine spring is adopted in various MEMS sensors [15,19].

The design target refers to ADXL327. ADXL327 is a high precision, low power, tri-axis accelerometer with signal conditioned voltage outputs from Analog Devices Inc. (ADI, Norwood, MA, USA). ADXL327 measures acceleration with a full-scale range of ±2 g, a range of 0.5 Hz to 1600 Hz for the *x*-and *y*-axis, and a range of 0.5 Hz to 550 Hz for the *z*-axis. In our design, the resonant frequency was targeted lower than 5 kHz and the sensing range was ±1 g. ADXL327 can measure both the static acceleration of gravity in tilt-sensing applications and dynamic acceleration resulting from motion, shock, or vibration. The main application is for navigation and motion detection [20].

In this paper, the design, simulation and mechanical characterization of the proposed CMOS/MEMS accelerometer is presented. The 0.18 μm application-specific integrated circuit (ASIC)-compatible CMOS/MEMS process was adopted for sensor and circuit implementation. While the circuit as well as the electrical characterization was presented in our previous works [21,22]. Section 2 describes the process flow and structure design of the accelerometer, while the theory and simulation were also analyzed. The approximate analytical model for the spring design was also proposed. Section 3 describes the measurement results of the proposed CMOS/MEMS accelerometer. Section 4 presents the discussion of the proposed accelerometer, a comparison of the performance with a state-of-the-art alternative and presents the conclusions of this work.

## 2. Materials and Methods

### 2.1. Process Flow

The proposed CMOS/MEMS accelerometer was implemented in the 0.18 μm ASIC-compatible CMOS/MEMS process. The integrated circuit (IC) foundries were Taiwan Semiconductor Manufacturing Company (TSMC, Hsinchu, Taiwan) 0.18 μm mixed-signal/radio frequency (RF) CMOS process with an Asia Pacific Microsystems, Inc. (APM, Hsinchu, Taiwan) MEMS post-process and United Microelectronics Corporation (UMC, Hsinchu, Taiwan) 0.18 μm mixed-signal/RF CMOS process with a UMC MEMS post-process.

The ASIC compatible 1P6M process started with a 0.18 μm standard CMOS process. The CMOS process consisted of one poly-silicon layer and six metal layers that can be used for wiring and circuit integration.

**Figure 1:**
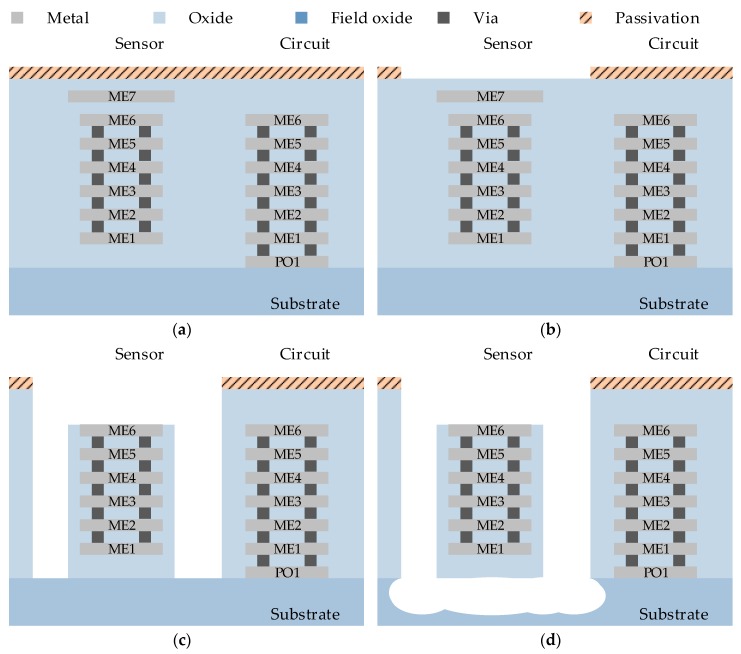
Cross-sectional view of the application-specific integrated circuit (ASIC)-compatible complementary metal-oxide semiconductor (CMOS)/micro-electromechanical system (MEMS) process flow: (**a**) The standard CMOS process with an additional patterned metal7 (ME7) layer; (**b**) The thick photoresist passivation layer is deposited for etch protection; (**c**) The anisotropic silicon oxide dry etching; (**d**) The isotropic silicon substrate dry etching.

The micromachining process was performed on the wafer of a standard CMOS process. The process flow is illustrated in Figure 1, where PO1 is the poly-silicon layer and metal1 (ME1) to metal6 (ME6) are the six metal layers. After the standard CMOS process, an additional patterned metal7 (ME7) layer and the passivation layers were deposited at the top of the structure and patterned as the etch-resistant mask (Figure 1a). A thick photoresist passivation layer was then deposited, which defined above the circuit and other regions for etch protection by using this mask, except the MEMS region (Figure 1b). The whole post-CMOS fabrication was performed using a dry etching processes. The area without photoresist protection was subjected to both anisotropic silicon oxide dry etching (Figure 1c) and isotropic silicon substrate dry etching (Figure 1d). The region without the photoresist passivation layer mask defined the MEMS etching region for the post-process. Metal 7 covered the whole microstructure to define the microstructures. The microstructures were released by isotropic silicon substrate dry etching. The passivation layer above the electronic circuits may have been slightly damaged after the post-micromachining process. The remaining photoresist layer was cleaned after the silicon etching.

### 2.2. Accelerometer Design

The single axis accelerometer was first implemented and the tri-axis accelerometer was later developed. The proposed CMOS/MEMS accelerometer consisted of a proof mass, sensing fingers, springs, and a curl matching frame.

#### 2.2.1. Single Axis Accelerometer

**Figure 2:**
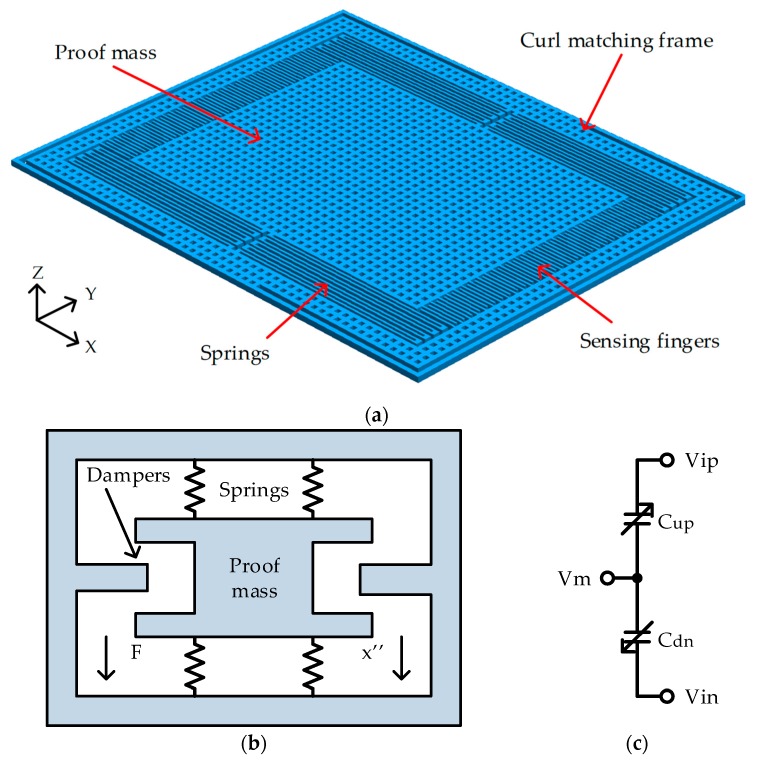
The proposed accelerometer: (**a**) The top view of the proposed accelerometer; (**b**) Mechanical model of the structure; (**c**) Circuit model of the structure.

Figure 2a is the top view of the proposed single axis accelerometer. The proof mass was suspended above the substrate by four sets of springs. The proof mass was a perforated structure that can be undercut etched to release the suspended structures. The density and size of the etching holes was limited by the etching condition of the undercut process. The design used a 6 μm × 6 μm etching hole and 6 μm spacing to form the proof mass based on the MEMS design rules from the manufacturers.

The micro-accelerometer was equivalent to the mechanical model in Figure 2b. It is a second-order mass-spring-damper system modeled by the force balance equation, where *F* is applied force, *m* is the mass of suspended proof mass, *x* is the displacement, *b* is the damping coefficient and *k* is the spring constant:

(1) $$F=mx^{\prime \prime }+bx^{\prime }+kx$$

The displacement (*x*) was transformed into capacitance (*ΔC*) by sensing fingers. The capacitance-to-voltage readout circuit transformed the capacitance to voltage. The circuit model in Figure 2c was simulated with a readout circuit. The circuit was simulated with Cadence Spectre simulator (Cadence Design Systems, Inc., San Jose, CA, USA).

The stiffness of the spring plays an important role in sensor design. Softer springs have less stiffness, and this means the device will have larger displacement, and hence larger capacitance (*ΔC*). The stiffness of the spring was decided by the width, length and turns of the springs.

Detailed models can be used to obtain more accurate results at the expense of speed of analysis. By developing a simplified analytical model, we gained insight regarding the mechanical behavior. Accurate results using elaborate models can be obtained using a finite element method (FEM) simulation. Simulations were carried out in CoventorWare 10 (Coventor, Inc., Cary, NC, USA).

MemMech is the FEM mechanical solver of CoventorWare, which is capable of computing displacement, reaction force and modal displacement. The material database provided contains characterized material properties for mechanical simulation. For a linear analysis, the displacement was calculated with the assumption that the stiffness is constant. For the nonlinear structural analysis in MemMech, the structure’s stiffness changed as it deformed. The stiffness matrix of the structure was much more complicated to solve than a linear analysis. The nonlinearity caused by material nonlinearity, boundary nonlinearity and geometric nonlinearity were considered in the FEM simulations.

**Figure 3:**
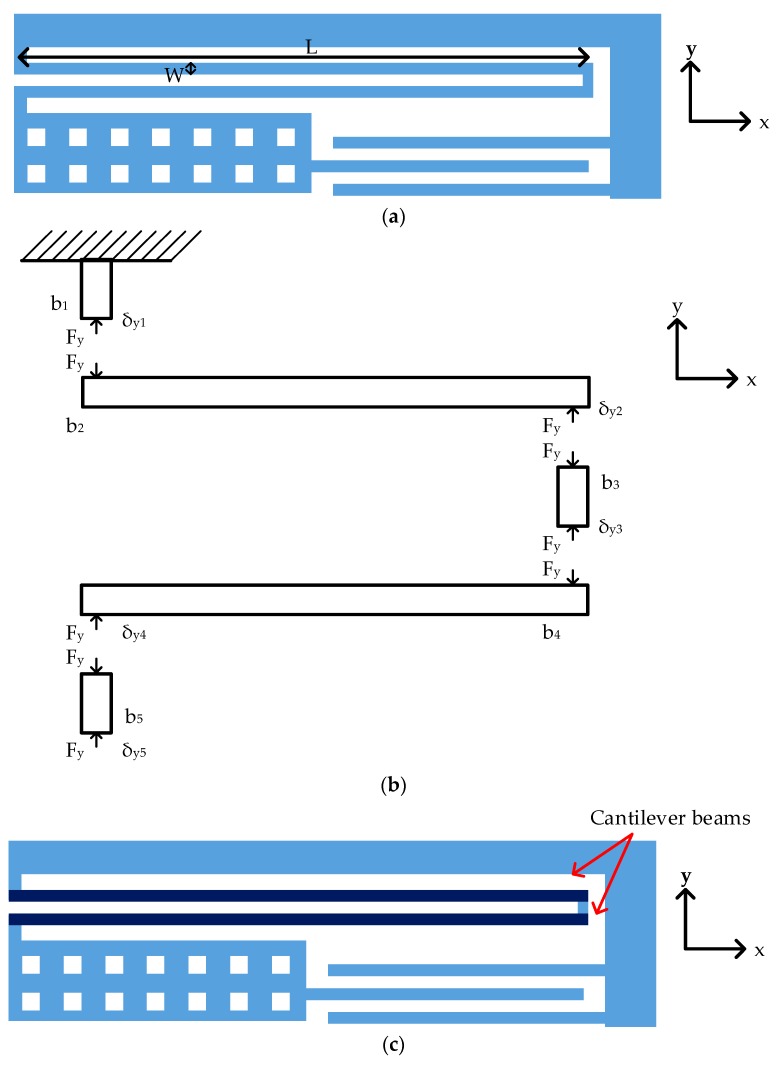
The schematic of serpentine spring: (**a**) Spring design parameters; (**b**) The free body diagram; (**c**) The proposed simplified model.

In this paper, serpentine springs are adopted for structure design, as in Figure 3a. By analyzing the structure, an approximate analytical model for the spring design is presented.

The schematic of proposed serpentine structure is shown in Figure 3a. Figure 3b shows the free body diagram of a serpentine spring. The beam segments were indexed from *b*_1_ to *b*_5_. The spring constant was found by applying a force balance to each beam segment. According to Hooke’s law, the relation between applied force (*F_y_*), spring constant along *y*-axis (*k_y_*) and displacement along *y*-axis (*δ_y_*) is formulated below:

(2) $$F_{y}=k_{y}\cdot \delta_{y}$$

As in Figure 3b, a lateral force along the *y*-axis (*F_y_*_)_ was applied at the end of the spring. The displacement along *y*-axis for each beam segment was given by:

(3) $$\delta_{yi}=\frac{F_{y}}{k_{yi}}$$

where *i* is the index of beam segment from 1 to 5, *δ_yi_* is the corresponding displacement along *y*-axis, and *k_yi_* is spring constant of the segment along *y*-axis.

The spring constant was obtained by summing the displacement of each segment and then divided by the applied force *F_y_*.

(4) $$\begin{array}{cc} \delta_{y} & =\sum_{i=1}^{5}\delta_{yi}\\ & =\delta_{y1}+\delta_{y2}+\delta_{y3}+\delta_{y4}+\delta_{y5}\\ & =\frac{F_{y}}{k_{y1}}+\frac{F_{y}}{k_{y2}}+\frac{F_{y}}{k_{y3}}+\frac{F_{y}}{k_{y4}}+\frac{F_{y}}{k_{y5}} \end{array}$$

Beam segment *b*_2_ and *b*_4_ were clamped-guided cantilever beams, hence spring constants *k_y_*_2_ and *k_y_*_4_ are listed below, where *k_c_* is spring constant along the *y*-axis, *E* was Young’s modulus of elasticity, *t* was the thickness of structure, *W* is the width of spring, *n* was the number of cantilever beam segments in series and *L* was the length of spring [23].

(5) $$k_{c}=\frac{EtW^{3}}{L^{3}}$$

Beam segments *b*_1_, *b*_3_ and *b*_5_ were rectangular beams hence spring constants *k_y_*_1_, *k_y_*_3_ and *k_y_*_5_ are given by *k_s_* [23]. The beam segments *b*_1_, *b*_3_ and *b*_5_ were very stiff along the *y*-axis. There was almost no displacement along the *y*-axis. The width of the segment was deliberately selected two times larger than the cantilever beam to minimize the displacement of segments *b*_1_, *b*_3_ and *b*_5_. The resulting spring constant was about 10^5^ times larger than *k_y_*_2_ and *k_y_*_4_.

(6) $$k_{s}=\frac{EtW}{L}$$

By ignoring the displacement of beam segments *b*_1_, *b*_3_ and *b*_5_, the serpentine spring only consisted of cantilever beams in series as in Figure 3c.

(7) $$\delta_{y}\approx \frac{F_{y}}{k_{y2}}+\frac{F_{y}}{k_{y4}}=\frac{2F_{y}}{k_{c}}$$

(8) $$k_{y}\approx \frac{F_{y}}{\delta_{y}}=\frac{F_{y}}{\frac{2F_{y}}{k_{c}}}=\frac{k_{c}}{2}$$

The spring constant of *n* cantilever beam segments in the *y*-axis was given by:

(9) $$k_{y}=\frac{k_{c}}{n}=\frac{EtW^{3}}{nL^{3}}$$

The whole structure consisted of four sets of serpentine structures. Therefore, the spring constant of whole structure in the *y*-axis was four times that of a single serpentine structure.

(10) $$k_{y}=\frac{4EtW^{3}}{nL^{3}}$$

Table 1 summarizes the spring design parameters of proposed single axis accelerometer.

The displacement along the *y*-axis (*δ_y_*) can be obtained by the following equation where *m* is mass of the proof mass and *a_y_* was the acceleration along *y*-axis. The 1 g acceleration *a_y_* was around 9.81 m/s^2^. The dimension of proof mass was 606 μm × 462 μm × 10.14 μm and *m* was around 4.32 μg.

(11) $$F_{y}=m\cdot a_{y}=k_{y}\cdot \delta_{y}$$

With mass and spring constant, the resonant frequency was given by:

(12) $$f=\frac{1}{2\pi }\sqrt{\frac{k_{y}}{m}}$$

Table 2 compares the results predicted by FEM simulations and the proposed simplified analytical model. The predicted spring constant was slightly higher than the FEM results since the displacement was underestimated. From the simplified analytical model above, the *y*-axis spring constant was proportional to *W*^3^, therefore the width of the spring (*W*), must be kept small to get higher sensitivity. The spring width (*W*), was limited to 4 μm by the CMOS/MEMS process. Increasing the spring length (*L*), or the number of cantilever beam segments in series *n* in a limited size can produce higher sensitivity. The proposed accelerometer had the displacement of 104.99 nm at 1 g. The FEM simulation results are listed in Table 3.

#### 2.2.2. Tri-Axis Accelerometer

**Figure 4:**
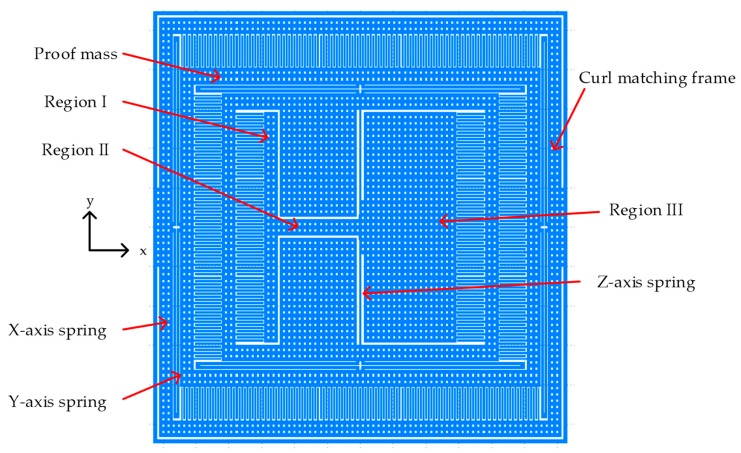
Top view of the proposed tri-axis accelerometer.

Figure 4 shows the proposed tri-axis single proof mass accelerometer. The tri-axis single proof mass accelerometer had an area of 1096 μm × 1256 μm. In order to suppress the structure curving effect, a curl matching frame was presented to achieve the same structure curling at the proof mass and the frame. The perforated structure and the layer combination were same for the proof mass and the frame to match the curling of the two parts. The layer combination ME1 and ME6 was chosen based on our previous work [9]. The *z*-axis sensor was embedded in the proof mass of the *y*-axis and the *y*-axis sensor was embedded in the proof mass of *x*-axis sensor. The springs of the *x* and *y*-axis were similar to a single axis design. Table 4 summarizes the in-plane (*x*-axis and *y*-axis) spring design parameters. Table 5 shows the results predicted by the FEM simulations and the proposed simplified analytical model.

**Figure 5:**
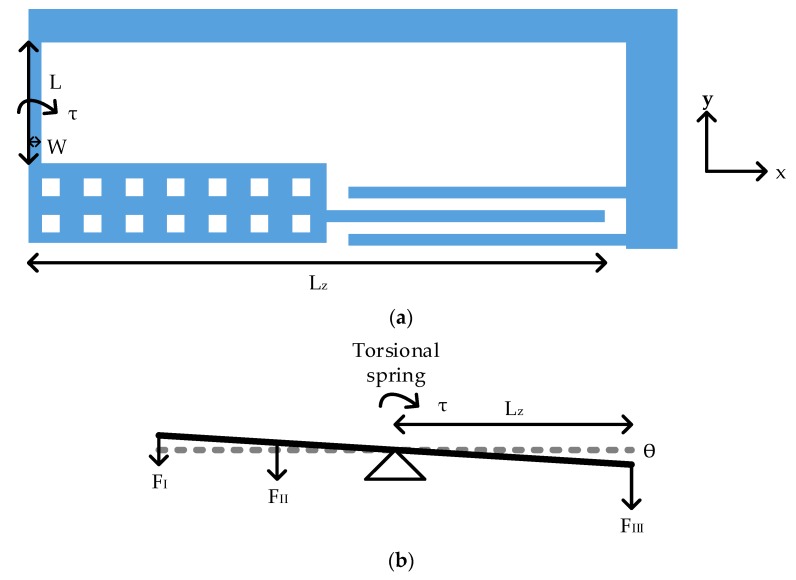
The schematic of torsion spring: (**a**) Spring design parameters; (**b**) The free body diagram.

The torsion spring in Figure 5a was adopted for out-plane sensing. The imbalanced torsional *z*-axis sensing element was embedded in the in-plane proof mass. The design equation of the torsion spring was given by [23,24]:

(13) $$k_{\theta }=\frac{GtW^{3}}{L}\left[\frac{1}{3}-0.21\frac{W}{t}\left(1-\frac{W^{4}}{12t^{4}}\right)\right]$$

where *G* is shear modulus, *W* is the width of the torsion beam, *L* is the length of the torsion beam, *t* is structure thickness. Table 6 summarizes the spring design parameters.

The whole structure consisted of two sets of torsional structures. Therefore, the spring constant of whole structure was two times that of a single torsional structure.

The imbalanced sensing element consisted of three regions as in Figure 4. The design parameters are specified in Table 7.

Figure 5b shows the free body diagram of a torsion spring. *F_I_* to *F_III_* are force from these three parts. According to Hooke’s law in angular form, the relation between applied torque (*τ*), torsion spring constant (*k_θ_*) and rotation angle (*θ*) is formulated below:

(14) $$\tau =k_{\theta }\theta$$

The displacement along *z*-axis (*δ_z_*) was obtained by the following equation where *L_z_* is the distance from the torsion spring to the sensing finger as in Figure 5. For 1 g acceleration *τ* was around 2.77 × 10^−12^ N·m, rotation angle was 3.23 × 10^−5^ rad and *δ_z_* was around 16.24 nm. The displacement of FEM simulation was 20.08 nm. The FEM simulation results are listed in Table 8.

(15) $$\delta_{z}=\theta \cdot L_{z}$$

## 3. Results

**Figure 6:**
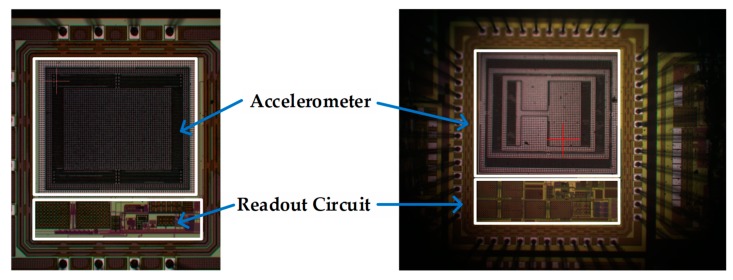
The chip die photo: (**a**) The single axis test chip; (**b**) The tri-axis test chip.

Figure 6 shows the chip photography for the sensors and readout circuitry. The single axis accelerometer had an area of 768 μm × 888 μm. The tri-axis accelerometer had an area of 1096 μm × 1256 μm.

### 3.1. Surface Topography Measurement

**Figure 7:**
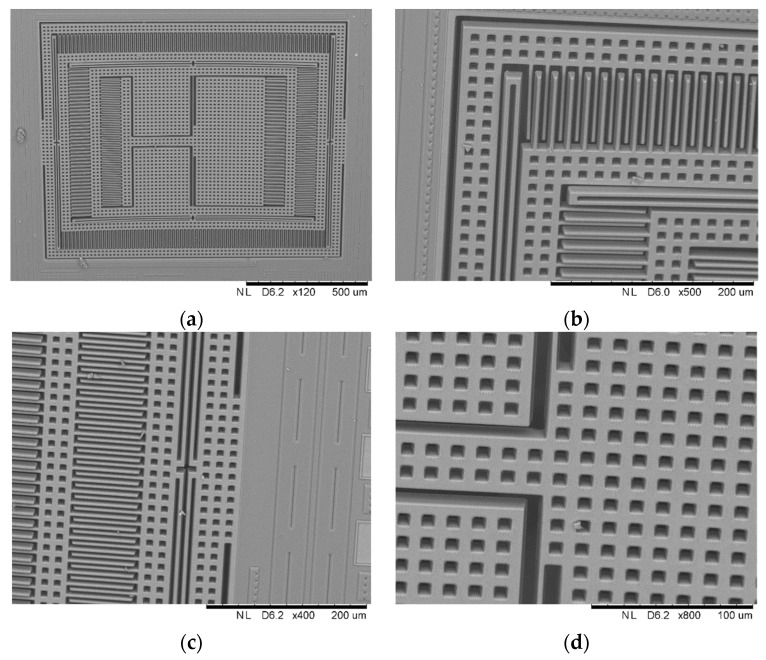
The SEM images of the proposed accelerometer: (**a**) The whole structure; (**b**) The curl matching frame; (**c**) The *x*-axis spring; (**d**) The torsion spring.

The scanning electron microscope (SEM, TM3000, Hitachi, Tokyo, Japan) image of the whole structure is shown in Figure 7a. Figure 7b shows the curl matching frame. The serpentine spring in Figure 7c is used for *x*-axis sensing. Figure 7d shows the fabricated torsion spring for *z*-axis sensing.

**Figure 8:**
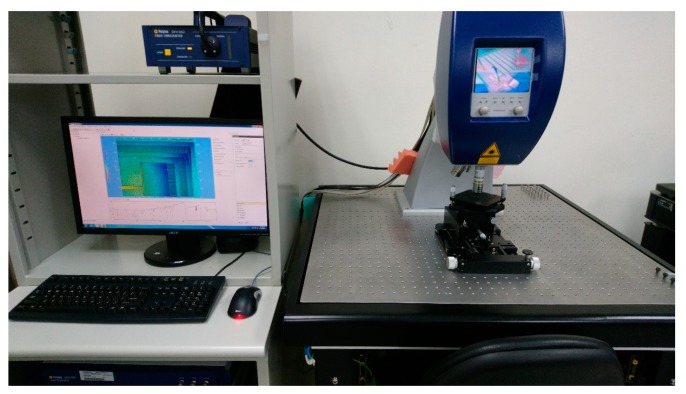
Measurement setup surface topography measurement.

The white light interferometer (MSA-500, Polytec, Waldbronn, Germany) was used for surface topography measurement as in Figure 8. The white light interferometry measured the surface height and constructed three-dimensional surface profiles.

**Figure 9:**
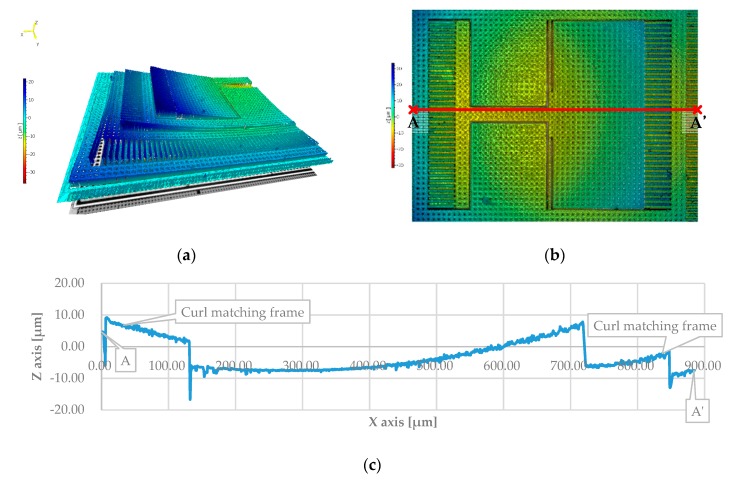
The surface profile of the proposed accelerometer: (**a**) The three-dimensional view; (**b**) The top view; (**c**) The cross-section view.

Figure 9a shows the three-dimensional view of the structure. The structure curling is around 20 μm. Figure 9b shows the top view of the structure and the curvature. A and A’ are located at the curl matching frame. Figure 9c shows the curvature of AA’ cross section. The curl matching frame had the same curvature as the structure which compensated the curl effect as illustrated in Figure 9c.

### 3.2. Mechanical Measurement

**Figure 10:**
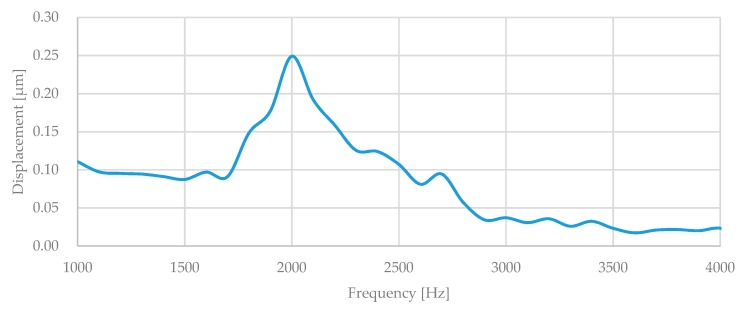
Frequency response of the proposed single axis accelerometer.

An in-plane vibration analyzer (MSA-500, Polytec, Waldbronn, Germany) was used to characterize the microstructure. The resonant frequency was detected optically at atmospheric pressure at room temperature with a laser Doppler vibrometer. Figure 10 shows the frequency response of the single axis accelerometer. The resonant was around 2 kHz. The in-plane resonant frequency of tri-axis accelerometer was around 2.5 kHz.

**Figure 11:**
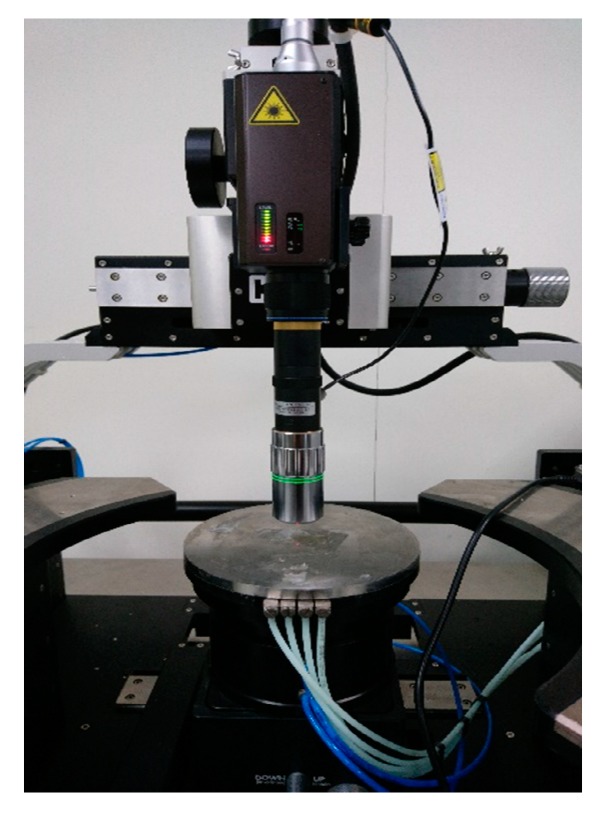
Measurement setup for out-plane vibration characterization.

**Figure 12:**
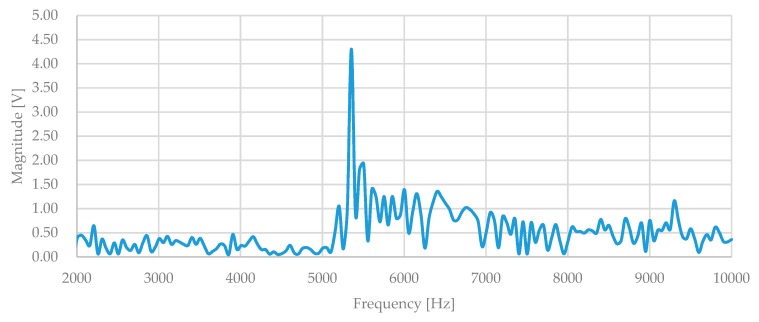
Out-plane frequency response of the proposed tri-axis accelerometer.

The out-plane (*z*-axis) motion was measured using a laser Doppler vibrometer (LV-1800, Ono Sokki, Yokohama, Japan). The output of the laser Doppler vibrometer was monitored using a network analyzer (Agilent 4395A, Keysight Technologies, Santa Rosa, CA, USA). Figure 11 shows the measurement setup for out-plane vibration characterization. The out-plane resonant frequency of tri-axis accelerometer was around 5.35 kHz, as in Figure 12.

## 4. Discussion and Conclusions

The tri-axis single proof mass accelerometer had an area of 1096 μm × 1256 μm, while the single axis accelerometer had an area of 816 μm × 696 μm. Comparing the three individual sensing units, the tri-axis single proof mass accelerometer reduces 23.77% of the chip area. To solve the curving problem, the layer combination for residual stress reduction and the curl matching frame are presented. For in-plane sensing, the serpentine spring is used for both single and tri-axis design. For out-plane sensing, the torsion spring is adopted. Table 9 compares the performance of proposed model, FEM simulation and experimental results.

For out-plane design, both of the models proposed and the FEM results show disagreement with the experimental results. The difference could be due to the dimensional variation of the process technology. The fabricated devices suffer from substantial parameter variations, from wafer to wafer and from lot to lot. Process variation of around 10% is usually considered in circuit design. The CMOS/MEMS process limits the minimum width of 4 μm. The 4 μm spring width was chosen to increase sensing capacitance. The design targets high sensitivity while process variation is anticipated. Spring width is the key design parameter to determine the spring constant and resonant frequency according to the proposed model. Geometries corresponding to 10% variation are depicted in Table 10. Increasing the spring width can lower the dimension variation at the cost of lowering sensitivity.

This paper presents the design, simulation and mechanical characterization of a proposed CMOS/MEMS accelerometer. In this study, two accelerometer designs were evaluated, both theoretically and experimentally. The monolithic CMOS/MEMS accelerometer was fabricated using the 0.18 μm ASIC-compatible CMOS/MEMS process. An approximate analytical model for the spring design was presented. Surface topography measurement adopted both scanning electron microscope and white light interferometer for three-dimensional surface profiles observation. Mechanical measurement was performed for both in-plane and out-plane vibration analysis. The experiments show that the resonance frequency of the proposed tri-axis accelerometer was around 5.35 kHz for out-plane vibration.

Table 11 compares the performance of the proposed tri-axis accelerometer to the state-of-the-art CMOS/MEMS capacitive accelerometers. The proposed accelerometer was fabricated using a 0.18 μm CMOS/MEMS process. Compared with [10], which is a single axis accelerometer design, the proposed tri-axis accelerometer had an area of 1096 μm × 1256 μm.

## Figures and Tables

**Table 1:** Spring design parameters.

Specifications	Design
Young’s Modulus of Elasticity (*E*) (GPa)	70
Spring Width (*W*) (μm)	4
Spring Length (*L*) (μm)	370
Cantilever Beam Segments (*n*) (count)	8
Structure Thickness (*t*) (μm)	10.14

**Table 2:** Comparisons of design and finite element method (FEM) simulation results.

Specifications	Design	FEM	Error (%)
*y*-axis Spring Constant (*k_y_*) (N/m)	0.45	0.41	8.18

**Table 3:** FEM simulation results.

Specifications	FEM
Displacement at 1 g (nm)	104.99
Initial Capacitance (*C*_0_) (fF)	91.97
Capacitance (*ΔC*) (fF)	2.35
Resonant Frequency (*f*_0_) (Hz)	1562.85
Mass (*M*) (μg)	4.32
Spring Constant (*K*) (N/m)	0.415

**Table 4:** In-plane spring design parameters.

Specifications	*x*-Axis	*y*-Axis
Young’s Modulus of Elasticity (*E*) (GPa)	70	70
Spring Width (*W*) (μm)	5	5
Spring Length (*L*) (μm)	472	489
Cantilever Beam Segments (*n*) (count)	2	2
Structure Thickness (*t*) (μm)	10.14	10.14

**Table 5:** Comparisons of design and finite element method simulation results.

Specifications	Design	FEM	Error (%)
*x*-axis Spring Constant (*k_x_*) (N/m)	1.69	1.63	3.45
*y*-axis Spring Constant (*k_y_*) (N/m)	1.52	1.50	1.19

**Table 6:** Torsion spring design parameters.

Specifications	Design
Shear Modulus (*G*) (GPa)	79
Spring Width (*W*) (μm)	4
Spring Length (*L*) (μm)	300
Structure Thickness (*t*) (μm)	10.14

**Table 7:** Proof mass design parameters.

Part	Area (μm × μm)	Moment Arm Length (μm)
Region I	700 × 40	280
Region II	260 × 40	130
Region III	700 × 30	150

**Table 8:** FEM simulation results.

Specifications	*x*-Axis	*y*-Axis	*z*-Axis
Displacement at 1 g (nm)	85.05	62.59	20.08
Resonant Frequency (*f*_0_) (Hz)	1708.45	1991.43	2634.14
Mass (*M*) (μg)	14.15	9.58	3.88

**Table 9:** Comparisons of proposed model, FEM simulation and experimental results.

Specifications	Proposed Model	FEM	Experimental Result
Single Axis: Resonant Frequency (*f*_0_) (Hz)	1575.20	1562.85	2000.00
Tri-axis: In-plane Resonant Frequency (*f*_0_) (Hz)	2036.05	1991.43	2500.00
Tri-axis: Out-plane Resonant Frequency (*f*_0_) (Hz)	3910.12	2634.14	5354.65

**Table 10:** Torsion spring constant and resonant frequency variation with spring width.

Specifications	Spring Width of 3.6 μm	Spring Width of 4 μm	Spring Width of 4.4 μm
Torsion Spring Constant (*k_θ_*) (N·m/rad)	6.45 × 10^−8^	8.57 × 10^−8^	1.10 × 10^−7^
Resonant Frequency (*f*_0_) (Hz)	3392.79	3910.12	4436.83

**Table 11:** Comparison of the proposed accelerometer to the state-of-the-art.

	[10]	[15]	[16]	[13]	[11]	This Work
Sensing Range (g)	±6	0.8~6	-	-	0.25~6.75	±1
Resonant Frequency (Hz)	4.7	9.54	-	1.7	5.27	5.35
Sensor Area (μm × μm)	430 × 600	-	500 × 500	-	-	1096 × 1256
Process	UMC 0.18 μm CMOS/MEMS	TSMC 0.35 μm 2P4M process	TSMC 0.35 μm 2P4M process	TSMC 0.35 μm CMOS	0.35 μm CMOS/MEMS	TSMC/UMC 0.18 μm CMOS/MEMS
